# Machine learning-based cell death marker for predicting prognosis and identifying tumor immune microenvironment in prostate cancer

**DOI:** 10.1016/j.heliyon.2024.e37554

**Published:** 2024-09-06

**Authors:** Feng Gao, Yasheng Huang, Mei Yang, Liping He, Qiqi Yu, Yueshu Cai, Jie Shen, Bingjun Lu

**Affiliations:** Department of Urology, Hangzhou Hospital of Traditional Chinese Medicine, Hangzhou, Zhejiang, 310007, China

**Keywords:** Prostate cancer, Programmed cell death, Machine learning, Tumor immune microenvironment, Biochemical recurrence

## Abstract

**Background:**

Prostate cancer (PCa) incidence and mortality rates are rising, necessitating precise prognostic tools to guide personalized treatment. Dysregulation of programmed cell death pathways in tumor suppression and cancer development has garnered increasing attention, providing a new research direction for identifying biomarkers and potential therapeutic targets.

**Methods:**

Integrating multiple database resources, we constructed and optimized a prognostic signature based on the expression of programmed cell death-related genes (PCDRG) using ten machine learning algorithms. Model performance and prognostic effects were further evaluated. We analyzed the relationships between signature and clinicopathological features, somatic mutations, drug sensitivity, and the tumor immune microenvironment, and constructed a nomogram. The expression level of PCDRGs were evaluated and compared.

**Results:**

Of 1560 PCDRGs, 149 were differentially expressed in PCa, with 34 associated with biochemical recurrence. The PCDRG-derived index (PCDI), constructed using the random forest algorithm, exhibited optimal prognostic performance, successfully stratifying PCa patients into two groups with significant prognostic differences. Patients with high PCDI scores exhibited poorer survival and lower immunotherapy benefit. PCDI was closely associated with the infiltration of specific immune cells, particularly positive correlations with macrophages and T helper cells, and negative correlations with neutrophils, suggesting that PCDI may influence the tumor immune microenvironment, thereby affecting patient prognosis and treatment response. PCDI was associated with age, pathological stage, somatic mutations, and drug sensitivity. The PCDI-based nomogram demonstrated excellent performance in predicting biochemical recurrence in PCa patients. Finally, the differential expression of these PCDRGs was verified based on cell lines and PCa patient expression profile data.

**Conclusion:**

This study developed an effective prognostic indicator for prostate cancer, PCDI, using machine learning approaches. PCDI reflects the link between aberrant programmed cell death pathways and disease progression and treatment response.

## 1 Introduction

Prostate cancer (PCa) represents one of the most significant health challenges in men worldwide, with its incidence and mortality rates exhibiting a concerning upward trend [1]. As an intricate disease, its progression and response to treatment can vary widely among individuals [2], underscoring the urgent need for personalized prognostic tools that can guide clinical decisions. Existing literature emphasizes the complexity of PCa biology and the critical need for advances in predictive modeling to improve patient outcomes [3]. The heterogeneity and diverse molecular features of PCa pose challenges for personalized treatment [4]. Therefore, a thorough understanding of the molecular mechanisms involved in the disease, the development of more accurate risk assessment models, and individualized treatment strategies are crucial for improving survival rates in PCa patients [5].

The concept of programmed cell death (PCD), including apoptosis, necroptosis, and pyroptosis, has gained substantial attention within cancer research, given its fundamental role in tumor suppression and cancer progression. Recent studies have highlighted the significant impact of dysregulation in PCD pathways on the prognosis of various cancers, including PCa [6]. This dysregulation offers a promising avenue for identifying biomarkers and therapeutic targets, making it a vital area of exploration in the quest to combat cancer more effectively [7]. Aberrant PCD not only contributes to the development of cancer but also impacts tumor sensitivity to radiation and chemotherapy drugs [8]. Therefore, an in-depth investigation of the molecular mechanisms regulating PCD and its role in tumorigenesis is crucial for developing novel diagnostic and therapeutic strategies [9]. In recent years, with a deeper understanding of different PCD pathways such as apoptosis, necroptosis, and pyroptosis, our comprehension of cancer pathogenesis has become more comprehensive, providing new opportunities for future personalized treatment [10].

Machine learning (ML) innovations revolutionize the utilization of extensive, complex biological data for developing highly accurate, clinically useful prognostic models. Distinct from conventional statistics, ML autonomously extracts features from intricate, non-linear data, minimizing assumption dependence [11]. In PCa, ML models have proven instrumental in the discovery of innovative biomarkers that significantly augment risk categorization and prognostic accuracy. For example, ML algorithms have been applied to analyze collagen-associated transcriptomic, proteomic, and metabolomic data from prostate tumor samples, yielding feature sets that demonstrate superior diagnostic performance for clinically significant PCa (csPCa) compared to conventional methods [12]. Integration of ML with magnetic resonance imaging (MRI) has shown promise in offering more precise diagnosis and stratification for PCa patients [13]. Moreover, machine learning algorithms have played a critical role in optimizing therapeutic regimens and evaluating the efficacy of novel agents in PCa management [14,15]. Recently, PCD-related prognostic signatures developed using ML have exhibited commendable performance in various cancer types. Additionally, ML algorithms have enabled the prediction of cancer cell susceptibility to PCD-inducing therapies by scrutinizing gene expression profiles [16], thereby facilitating personalized treatment strategies and enhancing the effectiveness of cancer interventions.

This research seeks to investigate the intersection of PCD and machine learning to built a prognostic signature for PCa. By focusing on PCD-related genes (PCDRGs), this study seeks to unveil novel biomarkers and construct an ML-based model that can accurately predict the biochemical recurrence (BCR) of PCa patients. The ultimate goal is to offer a more personalized prognosis, guiding treatment decisions and improving the quality of life for PCa patients. The significance of this research lies in its potential to contribute to personalized medicine, offering insights that could lead to more targeted and effective therapies for PCa patients.

## 2 Materials and methods

### 2.1 Data acquisition and processing

Clinical details and transcriptomic data of PCa patients were acquired from three databases: The Cancer Genome Atlas (TCGA, https://portal.gdc.cancer.gov), Gene Expression Omnibus (GEO, http://www.ncbi.nlm.nih.gov/geo), and cBioPortal (https://www.cbioportal.org/). A total of 708 samples were analyzed, including 321 from TCGA-PRAD, 247 from GSE116918, and 140 from MSKCC2010 (Table 1). All RNA-seq data were converted to Fragments Per Kilobase of transcript per Million mapped reads (FPKM) format. Prior to analysis, all data were log-transformed. Through a literature search [17], we included 19 patterns of PCD and 1560 unique PCDRGs in our analysis (Supplementary materials: Table S1).

**Table 1:** Clinical and pathological characteristics of the included study cohort.

characteristics	Cohorts		
	TCGA-PRAD (n = 321)	GSE116918 (n = 247)	MSKCC2010 (n = 140)
Age*	62 [57,66]	68 [63.5, 72]	NA
Status			
with BCR	62 (19.31)	56 (22.67)	36 (25.71)
without BCR	259 (80.69)	191 (77.33)	104 (74.29)
T stage			
T1	2 (0.62)	51 (20.65)	
T2	107 (33.33)	76 (30.77)	86 (61.43)
T3	212 (66.04)	92 (37.25)	47 (33.57)
T4	NA	4 (1.62)	7 (5)
Unknown	NA	24 (9.72)	
N stage			
N0	259 (80.69)	NA	NA
N1	62 (19.31)	NA	NA
M stage			
M0	320 (99.69)	NA	NA
M1	1 (0.31)	NA	NA

Abbreviation: BCR, biochemical recurrence; NA, not available.

Note: * indicates that the feature is represented by the median [interquartile range].

### 2.2 Machine learning

We employed an integrative approach, combining ten diverse machine learning algorithms (Table 2) and evaluating 101 algorithmic combinations [18,19]. A sequential methodology was implemented [18]: Univariate Cox regression analysis was utilized to pinpoint prognostic PCDRGs within the TCGA-PRAD dataset. Following this, a series of 101 algorithmic combinations were applied to these prognostic PCDRGs to develop predictive models using the leave-one-out cross-validation (LOOCV) method within the TCGA-PRAD dataset. Each developed model was subsequently evaluated using two independent validation datasets (GSE116918 and MSKCC2010). The performance of every model was evaluated by calculating Harrell's concordance index (C-index) across both validation datasets, with the model boasting the highest mean C-index being selected as the best performer. Detailed information can be found in the Supplementary Information. In accordance with previous descriptions in references [18,20], PCa patients were categorized into two groups—high PCDI and low PCDI—based on the cohort's median PCDI score. Next, the variations in BCR between the two groups was evaluated using Kaplan–Meier curves. Furthermore, calibration plots and Receiver Operating Characteristic (ROC) curves were constructed to evaluate the performance of PCDI.

**Table 2:** Machine learning algorithms selected for prognostic modeling.

Algorithm Name	Algorithm Features	Packages
Ridge Regression	Handles multicollinearity effectively; applies L2 regularization to prevent overfitting, suitable for high-dimensional genomic data.	glmnet
Lasso Regression	Utilizes L1 regularization for feature selection by shrinking coefficients to zero, aiding in identifying key DRLs affecting LUAD progression.	glmnet
Stepwise Cox Regression	Employs forward and backward selection to identify significant variables in survival analysis, ideal for assessing DRLs' impact on survival.	survival
CoxBoost	Adjusts for high-dimensional data through incremental fitting, enhancing predictive accuracy amidst multiple potential predictors.	CoxBoost
RSF	Random Survival Forest capable of handling censored data, excelling in managing complex interactions and non-linear relationships among DRLs.	randomForestSRC
Elastic Net (Enet)	Combines Ridge and Lasso properties, effective for correlated feature sets, offering balanced regularization and variable selection.	glmnet
plsRcox	Reduces dimensionality while preserving the relationship with survival outcomes, aiding in the examination of DRLs' influence on LUAD.	plsRcox
SuperPC	Enhances predictive accuracy by focusing on principal components strongly linked to survival, sharpening the analysis of DRLs' prognostic significance.	superpc
Survival-SVM	Adapts SVM for survival analysis, handling non-linear patterns, providing robust risk group classification.	survivalsvm
Gradient Boosting Machine (GBM)	Builds an ensemble of decision trees sequentially, improving accuracy iteratively for precise patient risk categorization based on DRL profiles.	superpc

### 2.3 Drug sensitivity analysis

The pRRophetic [21] package was utilized to analyze the sensitivity of patients in the TCGA-PRAD cohort to 45 different drugs, including “Axitinib”, “Bexarotene”, “Bicalutamide”, “Bleomycin”, and “Bortezomib”. Sensitivity assessment was performed using the pRRopheticPredict (.) function, with tissueType set to “all”, and batchCorrect using the ComBat method.

### 2.4 Differential expression and enrichment analysis

Raw transcriptomic count data were used for differential expression analysis. Subsequently, the “edgR” package was employed to identify differentially expressed PCDRG [22], applying a threshold of P < 0.05 and an absolute log2 fold change greater than 1. Enrichment analyses, including Gene Ontology (GO) and Kyoto Encyclopedia of Genes and Genomes (KEGG), were conducted using the clusterProfiler package [23]. Additionally, Gene Set Enrichment Analysis (GSEA) was carried out.

### 2.5 Immune infiltration evaluation

The Cancer Immunome Atlas (TCIA, https://tcia.at/) database was used to download the immunophenoscores (IPS) data of the patients in the TCGA-PRAD cohort [24]. Further, the IOBR [25] package was used for tumor microenvironment analysis, which includes various methods for assessing tumor immune infiltration.

### 2.6 Somatic mutation analysis

The maftools [26] package was adopted to analyze and visualize the somatic mutation data of the TCGA-PRAD cohort.

### 2.7 Nomogram construction and evaluation

We used univariate and multivariate Cox regression analyses to evaluate whether PCDI was associated with BCR when considering other clinical variables in PCa patients. Nomogram was constructed using the identified independent prognostic factors with p < 0.05 to predict the 1-, 2-, and 3-year BCR of PCa. The nomogram's performance was assessed through ROC, decision and calibration curve analyses.

### 2.8 Analysis of the expression of prognostic PCDRGs in PCa

In order to elucidate the expression patterns of PCDRGs in PCa, we retrieved their expression levels across PCa cell lines and normal cell lines from the Cancer Cell Line Encyclopedia (CCLE; accessible at https://depmap.org/portal/). Subsequently, we conducted a comparative analysis of PCDRG expression between normal tissue samples and cancerous tissue samples from The TCGA dataset to further delineate their expression profiles within the context of PCa.

### 2.9 Statistical analysis

All statistical analyses and data visualizations were performed using R Studio (version 4.2.3). Continuous data are described as the mean ± standard deviation (SD). Pearson correlation was applied to assess relationships between variables, and a p-value less than 0.05 was deemed indicative of statistical significance.

## 3 Results

### 3.1 Expression of PCRGs in PCa and their prognostic relevance

**Fig. 1:**
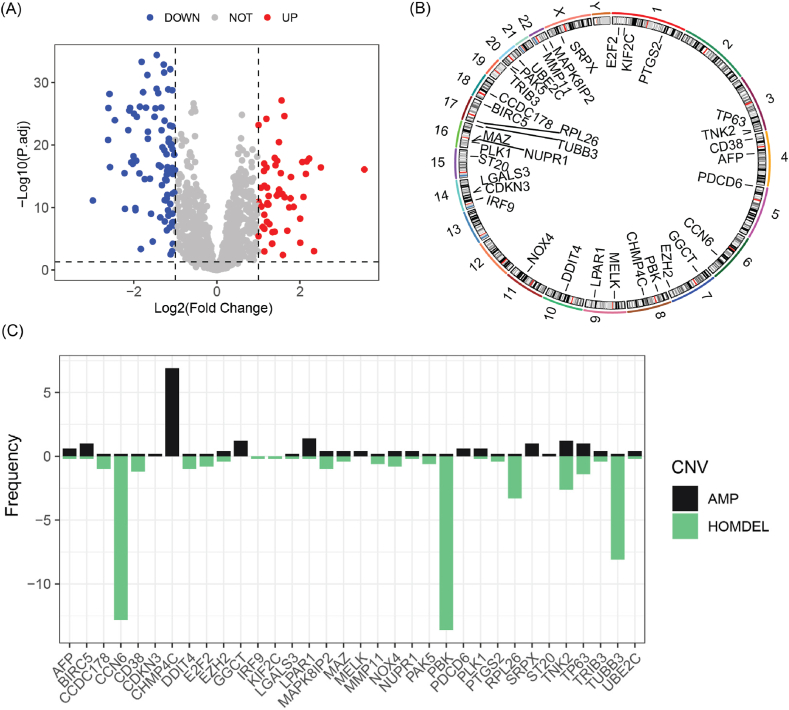
Expression and prognostic relevance analysis of PCDRGs in the TCGA-PRAD cohort. (A) Volcano plot of differentially expressed PCDRGs. (B) Chromosomal locations of prognostic-related PCRGs. (C) Copy number mutations of prognostic-related PCRGs. CNV, copy number variation; AMP, Amplification; HOMDEL, Homozygous Deletion.

**Fig. 2:**
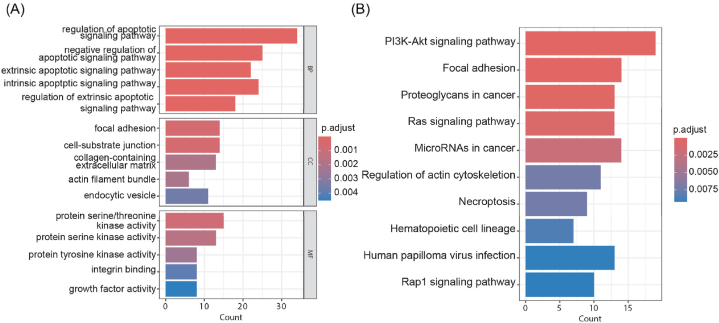
Barplots of the enriched terms of the differentially expressed PCDRGs in PCa. (A) Enriched GO terms of the differentially expressed PCDRGs. (B) Enriched KEGG pathways of differentially expressed PCDRGs.

Differential expression analysis revealed that 149 PCDRGs were aberrantly expressed in PCa, with 58 genes significantly upregulated and 91 significantly downregulated (Fig. 1A). Furthermore, we found that 34 of these differentially expressed genes (DEG) were linked to the BCR of PCa (Supplementary materials: Table S2). Fig. 1B displays the chromosomal locations of these BCR-related PCDRGs. Notably, among these genes, PBK (13.6 %), CCN6 (12.8 %), and TUBB3 (8.1 %) were identified as the top three harboring the most significant copy number deletions. In contrast, CHMP4C (6.9 %), LPAR1 (1.4 %), and GGCT (1.2 %) emerged as the foremost genes characterized by copy number amplifications, as depicted in Fig. 1C. Moreover, these PCDRGs were enriched in cell apoptosis processes and possess protein kinase activity (Fig. 2A). Furthermore, these genes are also involved in the PI3K-AKT signaling pathway, Ras signaling pathway, necroptosis, and Rap1 signaling pathway (Fig. 2B).

### 3.2 Construction and evaluation of the risk Feature-PCDI

**Fig. 3:**
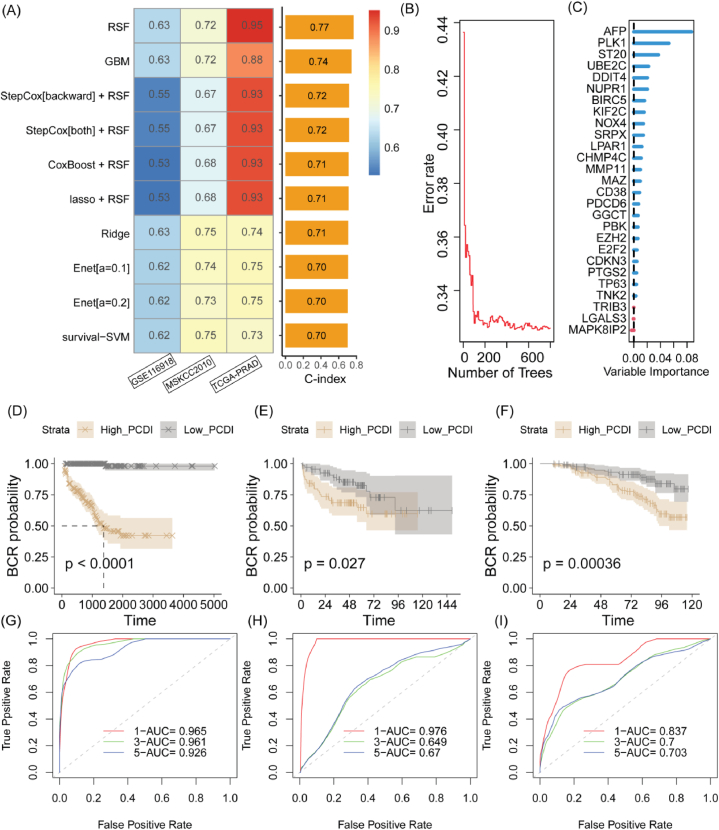
Construction of the PCa prognostic indicator PCDI using various machine learning algorithms. (A) Performance evaluation of PCDI construction based on combinations of 101 machine learning algorithms. (B) Prediction error rates. (C) The VIMP plot. (D–F) Survival analysis in the TCGA-PRAD, GSE116918, and MSKCC2010 cohorts. (G–I) ROC curve of PCDI in the TCGA-PRAD, GSE116918, and MSKCC2010 cohorts. RSF, random survival forest; GBM, gradient boosting machines; LASSO, least absolute shrinkage and selection operator; SVM, support vector machine; AUC, area under the curve.

We employed 10 ML algorithms to develop PCDI, and the optimal model were was selected based on the highest average C-index across the TCGA-PRAD, GSE116918 and MSKCC2010 cohorts (Fig. 3A and Supplementary materials: Table S3). Ultimately, our selection favored the RSF model grounded in 27 PCDRGs (Table 3, Supplementary materials: Fig. S1), owing to its preeminence as measured by the highest average c-index across the examined models. The evolution of 200 survival trees revealed a trajectory characterized by consistently subdued prediction error rates, affirming the model's resilience and reliability (Fig. 3B). Post the culmination of constructing 800 trees, we derived Variable Importance Measures (VIMP) for each feature instrumental in the formation of these trees. A heightened VIMP score is indicative of a gene's augmented influence on the prediction of BCR. As shown in Fig. 3C, the five highest-ranking genes were AFP, PLK1, ST20, UBE2C, and DDIT4. It was demonstrated that the High PCDI group experienced worse outcomes compared to the Low PCDI group in the TCGA-PRAD (p < 0.0001), GSE116918 (P = 0.00036), and MSKCC2010 (P = 0.027) cohorts (Fig. 3D–F). In the training cohort, the PCDI achieved an Area Under the Curve (AUC) of 0.965, 0.961, and 0.926 for predicting 1-, 3-, and 5-year BCR of PCa, respectively (Fig. 3G). For the MSKCC2010 cohort, the AUC values were 0.837, 0.7, and 0.703 (Fig. 3H). In the GSE116918 cohort, the corresponding AUC values were 0.976, 0.649, and 0.67 (Fig. 3I).

**Table 3:** The prognostic properties of 27 PCDI-related genes in prostate cancer.

Symbol	Name	HR	Lower95	Upper95	p-value	property
CD38	CD38 molecule	0.79	0.66	0.94	0.007	Anti-oncogenic
LGALS3	galectin 3	0.79	0.62	0.99	0.041	Anti-oncogenic
LPAR1	lysophosphatidic acid receptor 1	0.70	0.49	0.99	0.045	Anti-oncogenic
PTGS2	prostaglandin-endoperoxide synthase 2	0.75	0.61	0.93	0.007	Anti-oncogenic
SRPX	sushi repeat containing protein X-linked	0.74	0.55	0.99	0.041	Anti-oncogenic
TP63	tumor protein p63	0.78	0.62	0.98	0.031	Anti-oncogenic
AFP	alpha fetoprotein	4.43	2.09	9.40	0.000	Pro-oncogenic
BIRC5	baculoviral IAP repeat containing 5	1.76	1.34	2.32	0.000	Pro-oncogenic
CDKN3	cyclin dependent kinase inhibitor 3	1.91	1.33	2.75	0.000	Pro-oncogenic
CHMP4C	charged multivesicular body protein 4C	1.71	1.19	2.46	0.004	Pro-oncogenic
DDIT4	DNA damage inducible transcript 4	1.33	1.01	1.74	0.041	Pro-oncogenic
E2F2	E2F transcription factor 2	2.31	1.11	4.81	0.024	Pro-oncogenic
EZH2	enhancer of zeste 2 polycomb repressive complex 2 subunit	2.34	1.46	3.73	0.000	Pro-oncogenic
GGCT	gamma-glutamylcyclotransferase	1.51	1.12	2.05	0.007	Pro-oncogenic
KIF2C	kinesin family member 2C	2.03	1.37	3.00	0.000	Pro-oncogenic
MAPK8IP2	mitogen-activated protein kinase 8 interacting protein 2	1.46	1.08	1.98	0.014	Pro-oncogenic
MAZ	MYC associated zinc finger protein	1.85	1.18	2.88	0.007	Pro-oncogenic
MMP11	matrix metallopeptidase 11	1.85	1.46	2.35	0.000	Pro-oncogenic
NOX4	NADPH oxidase 4	2.45	1.36	4.42	0.003	Pro-oncogenic
NUPR1	nuclear protein 1, transcriptional regulator	1.70	1.22	2.37	0.002	Pro-oncogenic
PBK	PDZ binding kinase	1.52	1.09	2.12	0.013	Pro-oncogenic
PDCD6	programmed cell death 6	2.99	1.33	6.72	0.008	Pro-oncogenic
PLK1	polo like kinase 1	2.31	1.55	3.43	0.000	Pro-oncogenic
ST20	suppressor of tumorigenicity 20	4.30	2.16	8.55	0.000	Pro-oncogenic
TNK2	tyrosine kinase non receptor 2	2.11	1.30	3.42	0.003	Pro-oncogenic
TRIB3	tribbles pseudokinase 3	1.46	1.02	2.08	0.038	Pro-oncogenic
UBE2C	ubiquitin conjugating enzyme E2 C	1.55	1.25	1.93	0.000	Pro-oncogenic

### 3.3 PCDI was associated with drug sensitivity and tumor microenvironment

**Fig. 4:**
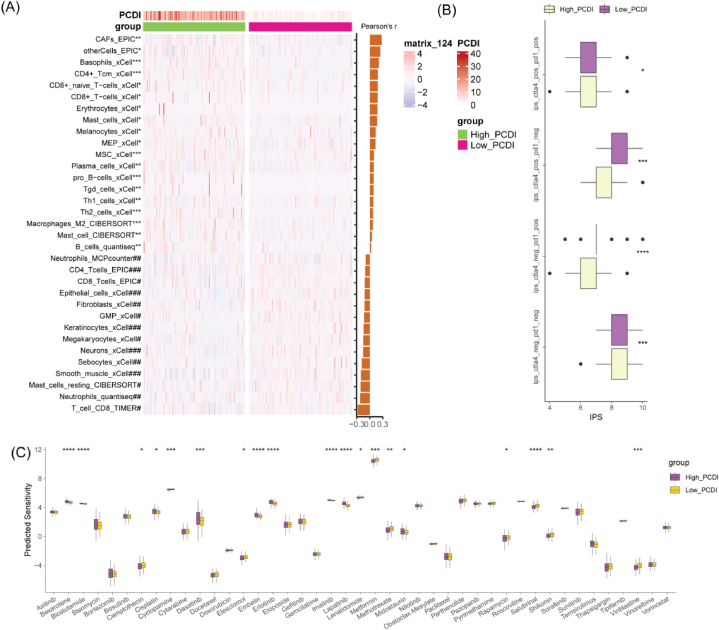
Association between PCDI and the TIME and chemotherapy response. (A) Correlation analysis between PCDI and tumor immune cell infiltration. (B) Differences in the IPS between the different PCDI groups. (C) Comparison of sensitivity to 45 chemotherapy drugs between the different PCDI groups. *p < 0.05, **p < 0.01, ***p < 0.001, ****p < 0.0001.

To comprehensively evaluate the association between PCDI and the tumor immune microenvironment (TIME), we systematically assessed immune cell infiltration using the IOBR package. Our data indicated that PCDI was associated with the infiltration of multiple immune cells types, primarily exhibiting positive correlations with macrophages, Th1 and Th2 cells, B cells, and other immune cells, while negatively correlating with neutrophils, sebocytes, megakaryocytes, and other cells. However, the correlation between PCDI and the presence of T cells and fibroblasts varied across different algorithms (Fig. 4A). The Low_PCDI group exhibited a higher IPS compared to the High_PCDI group (Fig. 4B), indicating higher immune cell infiltration and activation levels, suggesting better prognosis and response to immunotherapy. Additionally, PCDI and its constituent genes were significantly associated with drug sensitivity (Supplementary materials: Fig. S2). There were notable differences in sensitivity to 19 drugs between the different PCDI groups, including bexarotene, bicalutamide, and cisplatin (Fig. 4C).

### 3.4 Correlation of PCDI with clinicopathological features and somatic mutation profiles

**Fig. 5:**
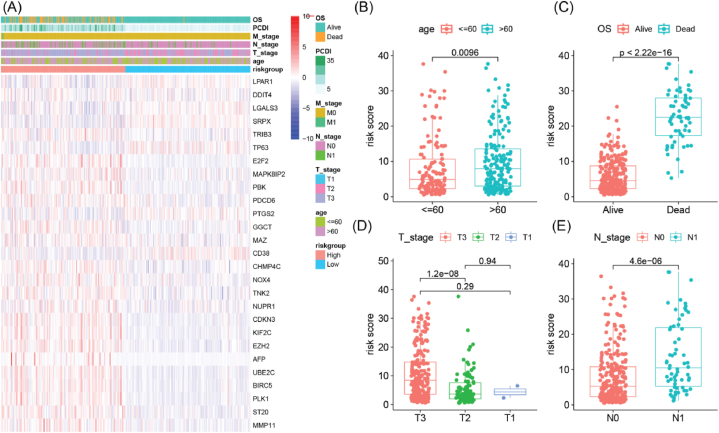
Correlation of PCDI with clinicopathological features and somatic mutation profiles. (A) Heatmap visualization of the PCDI constituent genes and clinical characteristics annotations. (B–E) Comparison of PCDI among subgroups with different age, survival outcome, T stage, and N stage. OS, overall survival.

We compared the expression of PCDI constituent genes and clinical characteristics between the two groups (Fig. 5A). The results indicated significant differences between the two groups in terms of survival outcome, PCDI, N stage, T stage, and age. The older age group had higher PCDI compared to the younger age group (Fig. 5B). Deceased patients had higher PCDI than alive patients (Fig. 5C). Patients with T3 stage had higher PCDI than those with T2 stage (Fig. 5D), and patients with N1 stage had higher PCDI than those with N0 stage (Fig. 5E). These findings underscored the association between age and clinical stage with the PCDI, revealing that patients of elder age and advanced stage exhibit elevated PCDI.

**Fig. 6:**
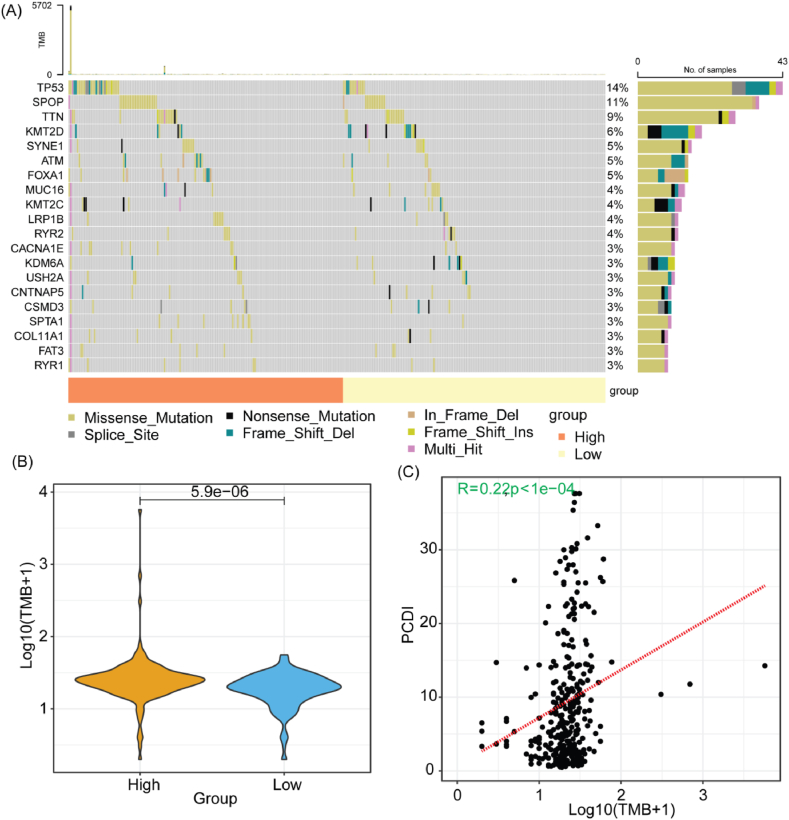
Analysis of the correlations between PCDI and somatic mutations in the TCGA-PRAD cohort. (A) Oncoplot showing the differences in the most frequently mutated genes between the two PCDI groups. (B) Differences in the TMB between the two PCDI groups. *p < 0.05. (C) Correlation analysis illustrating a weak positive correlation between PCDI and TMB. TMB, tumor mutational burden.

Fig. 6A displays the top 20 gene with the highest mutation frequency in the TCGA-PRAD cohort, with significant differences in the mutation rates of TP53, FOXA1, CACNA1E, and SPTA1 between the two groups. The patients in the High PCDI group showed a higher tumor mutational burden (TMB) compared to the Low_PCDI group (Fig. 6B). We found a weak positive correlation (r = 0.22) between PCDI and TMB, which was statistically significant (p < 1 × 10−4, Fig. 6C). The results illuminated the relationship between PCDI and somatic mutations, demonstrating that patients harboring a higher frequency of genomic alterations present with augmented PCDI.

### 3.5 Correlation of PCDI with gene expression regulation

**Fig. 7:**
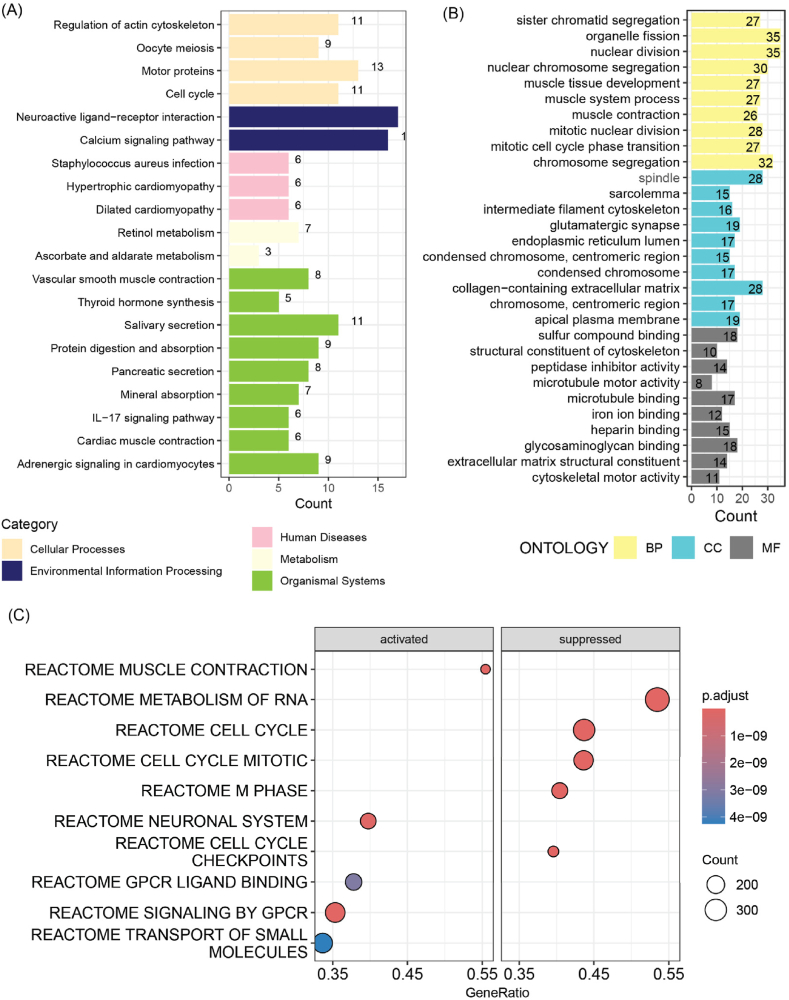
Differences in GO terms and pathways between the two PCDI groups. (A) Barplot of the enriched KEGG pathways of the DEGs between the two groups. (B) Barplot of the enriched GO terms of DEGs between the two groups. (C) Dotplot of the significantly activated or suppressed pathways in the High_PCDI group. BP, biological process; CC, cellular component; MF, molecular function.

As illustrated in Fig. 7A, the differentially expressed genes between the two risk groups were primarily enriched in the calcium signaling pathway, neuroactive ligand-receptor interaction, cell cycle, and IL-17 signaling pathway. Additionally, these genes were also involved in multiple GO terms, including organelle fission, nuclear division, mitotic cell cycle phase transition, and muscle tissue development (Fig. 7B). GSEA indicated that pathways such as GPCR ligand binding and neuronal systems were significantly activated in the High_PCDI group, whereas pathways related to RNA metabolism, the cell cycle, M phase, and cell cycle checkpoints were notably suppressed (Fig. 7C).

### 3.6 Construction of a nomogram for PCa based on PCDI

**Fig. 8:**
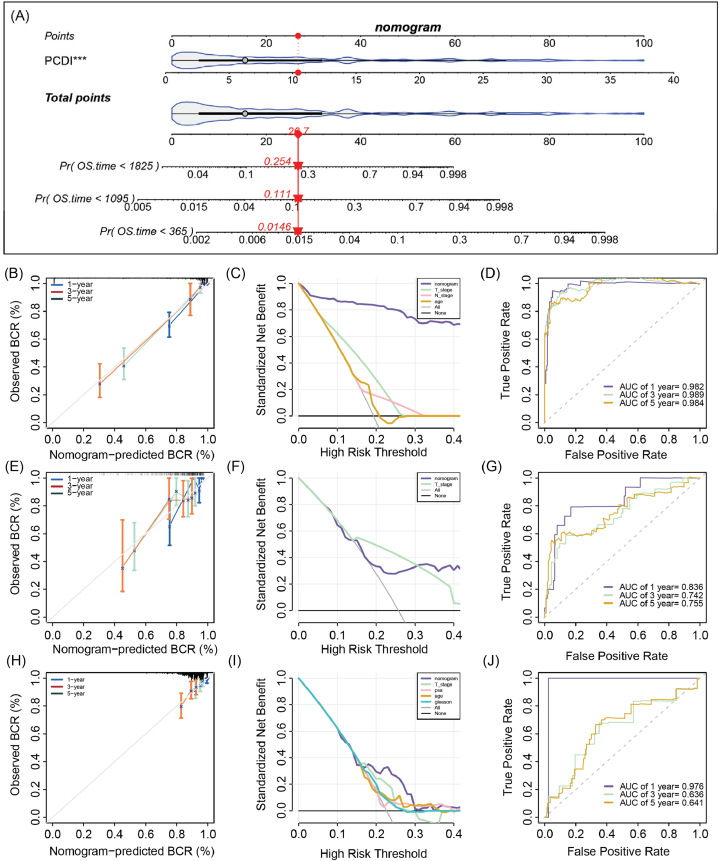
Development of a Nomogram for Prostate Cancer Patients Based on PCDI. (A) Nomogram developed using PCDI to predict the BCR for PCa patients. (B–D) Calibration plots and ROC curves showing the accuracy of the nomogram across the TCGA-PRAD, GSE116918, and MSKCC2010 cohorts. (E–G) Decision curve Calibration curve of the nomogram in the TCGA-PRAD, GSE116918 and MSKCC2010 cohorts. (H–J) ROC curve of the nomogram in the TCGA-PRAD, GSE116918 and MSKCC2010 cohorts. BCR, biochemical recurrence, AUC, area under the curve.

Upon conducting Univariate Cox Regression Analysis, we observed that the PCDI and T stage were significantly associated with BCR across all cohorts. N stage and PSA levels exhibited a significant correlation with BCR only in single cohort, a finding potentially attributable to the absence of these clinical parameters in other datasets due to limitations in data availability. In the subsequent Multivariate Cox Analysis, the PCDI was unequivocally identified as an independent prognostic factor in three distinct cohorts, underscoring its universality and robust predictive capability for BCR in patients diagnosed with PCa (Table 4). To assess the BCR in PCa patients, we developed a nomogram utilizing the PCDI (Fig. 8A). The model's predictive accuracy for these time points was validated using a calibration curve (Fig. 8B–D). When compared to alternative prognostic indicators, our nomogram demonstrated a superior standardized net benefit specifically for the 1-year BCR prediction (Fig. 8E–G). Furthermore, the nomogram's performance within the TCGA-PRAD cohort was evaluated using the AUC, resulting in scores of 0.982, 0.989, and 0.984, respectively (Fig. 8H). The nomogram's discriminative ability for predicting 1-, 3-, and 5-year BCR within the GSE116918 cohort was assessed, achieving AUC values of 0.835, 0.742, and 0.755, respectively (Fig. 8I). Similarly, in the MSKCC2010 cohort, the AUCs for forecasting 1-, 3-, and 5-year BCR probabilities were 0.976, 0.636, and 0.641, respectively (Fig. 8J). These results collectively underscored the nomogram's robust predictive capabilities across multiple cohorts, albeit with varying degrees of precision.

**Table 4:** Univariate and multivariate Cox regression analysis identified the prognostic value of PCDI in the PCa patients.

Variables	TCGA-PRAD		GSE116918		MSKCC2010	
**Univariate Cox**						
PCDI	1.3 [1.2–1.3]	7.40E-35	1.1 [1–1.1]	0.0012	1.2 [1.1–1.2]	1.20E-07
age	1 [0.99–1.1]	0.12	0.98 [0.94–1]	0.23	NA	NA
T stage	0.2 [0.085–0.46]	0.00015	1.3 [1–1.6]	0.03	3.4 [2.1–5.4]	7.00E-07
N stage	0.45 [0.27–0.77]	0.0035	NA	NA	NA	NA
PSA	NA	NA	1 [1–1]	0.032	NA	NA
Gleason.grade	NA	NA	1.2 [0.97–1.6]	0.089	NA	NA
**Multivariate Cox**						
PCDI	1.28 [1.23–1.34]	<0.001	1.07 [1.02–1.13]	0.01	1.13 [1.07–1.19]	<0.001
age	1.03 [0.98–1.08]	0.261	0.97 [0.93–1.01]	0.145	NA	NA
T stage	0.58 [0.24–1.39]	0.221	1.20 [0.94–1.53]	0.145	3.20 [1.89–5.40]	<0.001
N stage	0.95 [0.55–1.64]	0.856	NA	NA	NA	NA
PSA	NA	NA	1.00 [0.99–1.01]	0.401	NA	NA
Gleason.grade	NA	NA	1.10 [0.84–1.44]	0.477	NA	NA

Abbreviation: PCDI, PCDRG-derived index; PSA, Prostate-Specific Antigen; NA, not available.

### 3.7 Expression analysis of prognostic MTGs in PCa

**Fig. 9:**
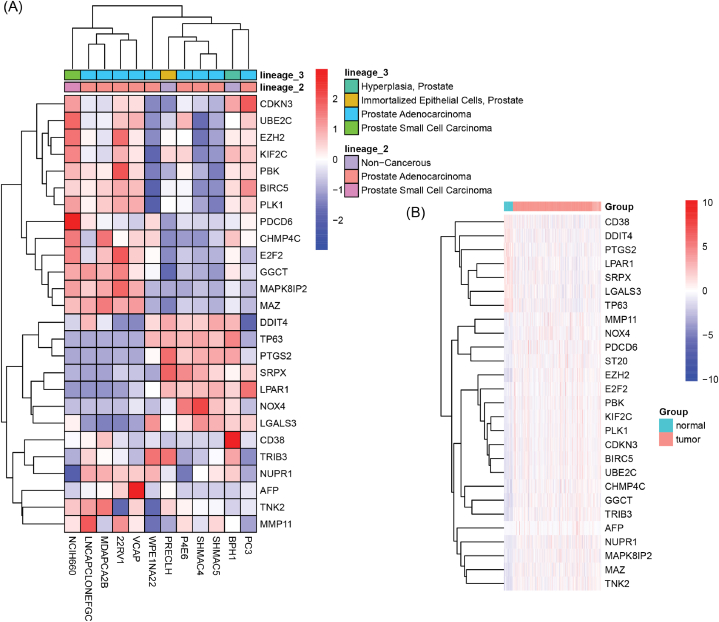
Validation of PCDRG expression in PCa. (A) Heatmap representation of the expression levels of the 26 PCDRGs across different prostate cell lines. (B) Heatmap depicting the expression patterns of the 27 PCDRGs within the TCGA-PRAD dataset.

The identification of PCa patients with BCR based on the prognostic features of PCDRGs represents a promising predictive strategy. Nevertheless, the potential association between the progression of PCa and the expression patterns of these prognostic PCDRGs has yet to be conclusively elucidated. Analysis of gene expression profiles across various PCa cell lines within the CCLE database suggests that multiple PCa cell lines, such as NCIH660, LNCAPCL0NEFGC, MDAPCA2B, 22RV1, VCaP, among others, display contrasting expression traits relative to non-cancerous cell lines like BPH-1 and PRECLH, with expression patterns that align similarly to those seen in the TCGA-PRAD dataset. In comparison to normal tissues, genes highly expressed in PCa tissues (with the exception of CD38) cluster together (Fig. 9A and B, Supplementary materials: Table S4). These findings suggested that the PCDRGs could have a significant impact on the development and progression of PCa.

## 4 Discussion

This study combines the mechanisms of programmed cell death with machine learning techniques to explore personalized prognosis and the benefits of immunotherapy in PCa. The research reveals the crucial roles played by genes associated with dysfunctional cell death pathways in PCa. By mining large biological datasets and employing advanced machine learning methods, a risk indicator called PCDI based on 27 key genes was established. This indicator successfully distinguishes different prognostic groups of PCa patients and is linked to the BCR, immune cell infiltration levels, drug sensitivity, and clinicopathological features. The study found that patients with higher PCDI exhibited poorer prognosis and lower response to immunotherapy, and were associated with higher frequencies of somatic mutations such as TP53 and FOXA1, as well as higher tumor mutational burden. The prognostic nomogram constructed based on PCDI can accurately predict patients' 1- to 5-year BCR risk, demonstrating good clinical utility and predictive performance.

Regarding the PCDI, five genes—AFP, PLK1, ST20, UBE2C, and DDIT4—exert a substantial influence. AFP, the first identified oncoprotein, not only serves as a potent prognostic biomarker in hepatocellular carcinoma screening but also plays a critical role in tumor progression by modulating immunoregulatory pathways [27]. A recent study has demonstrated that baseline AFP levels can predict the efficacy of immune checkpoint inhibitor therapy in advanced gastric cancer patients [28]. Prior studies have uncovered the antitumor activity of AFP-derived peptides against PCa [29], underscoring the importance of further elucidating AFP's role in the diagnosis and treatment of PCa. PLK1, a multifunctional serine/threonine protein kinase, is crucial for various cellular processes, including DNA replication, chromosome segregation, and stress response regulation [30]. Consistent with our findings, a wealth of previous research has solidified PLK1's status as a key oncogenic driver, with its inhibition leading to mitotic arrest and a pronounced antitumoral effect [31]. Specifically, in androgen-independent PCa cells, PLK1 expression is markedly elevated, and its suppression triggers necrotic cell death [32]. Moreover, evidence suggests that PLK1 inhibition potentiates the clinical outcomes of therapies targeting castration-resistant PCa (CRPC) [[33], [34], [35]]. Consequently, the current and future research emphasis lies in the development of novel PLK1 inhibitors, aiming to expand the therapeutic arsenal for PCa with efficacious and targeted treatment options.

ST20, also known as HCCS-1, exhibits downregulated expression in a variety of human malignancies, potentially fulfilling the role of a tumor suppressor gene by activating apoptotic signaling pathways, thereby restraining the progression of cervical cancer [36]. In contrast, our findings unveil an aberrantly heightened expression of ST20 in PCa, coupled with a strong association with adverse patient prognosis. This observation suggests that ST20 may adopt an antagonistic function within the pathophysiology of PCa, possibly operating as an oncogenic driver that facilitates tumor proliferation and invasiveness. The UBE2C oncogene is characterized by overexpression across numerous solid tumor types, inclusive of lethal CRPC, under the regulatory influence of the phosphorylation mediator complex subunit 1 [37]. A spectrum of *anti*-PCa agents has been shown to exert their effects through direct or indirect suppression of UBE2C, underscoring its significance as a therapeutic target [38,39]. DDIT4, predominantly recognized for its function in inhibiting the proliferative signaling pathway by suppressing the mTOR regulator within this cascade, thereby modulating metabolism [40], also emerges as a pivotal effector of autophagy. Through its regulation of lysosomal formation, DDIT4 contributes to the resistance of PCa cells to proteasome inhibitors, such as bortezomib [41]. Recent investigations have revealed that DDIT4 is subject to FTO-mediated N6-methyladenosine modification, implicating its involvement in PCa initiation and metastasis [42]. Therefore, elucidating their mechanisms of action can aid in exploring new diagnostic and therapeutic targets.

The PCDI captures the expression patterns of PCRGRs. A high PCDI level indicates poor prognosis for PCa patients. The observation aligns with prior study, which has shown that dysregulation of PCD processes is correlated with tumor development, progression, and treatment resistance [43,44]. Notably, PCDI exhibits a complex pattern of associations with various immune cell infiltrations. On one hand, PCDI is significantly positively correlated with tumor-associated macrophages (TAM) in an M2-polarized state. These macrophages can release pro-angiogenic factors (such as VEGF), facilitating the formation of new blood vessels for tumor nourishment and growth [45]. They can also secrete cytokines and metalloproteinases, enabling tumor cells to avoid immune detection and establishing an immunosuppressive environment that supports tumor cell growth and metastasis [46,47]. Furthermore, the significant correlation between PCDI and Th1/Th2 cell infiltration suggests that modulating the Th1/Th2 balance is an important strategy for cancer immunotherapy [48,49]. On the other hand, PCDI is inversely related to the infiltration of cells such as neutrophils, which promote tumor progression, potentially reflecting another aspect of PCDI's regulation of the tumor microenvironment [50]. Overall, PCDI may influence tumor development and prognosis by modulating the infiltration of TME immune cells, and the interplay between PCD processes and the tumor immune microenvironment is an area worthy of further exploration, as it may reveal new mechanisms underlying tumor development and provide new targets and strategies for tumor immunotherapy.

Drug resistance is a critical determinant of patient outcomes in oncology. Although the PCDI does not exhibit high correlations with sensitivity to most drugs, it comprises genes that have significant associations with drug sensitivity in PCa. Embelin, an active component derived from traditional herbal medicine, exerts antitumor effects on human PCa cells and significantly enhances the suppression of PCa by radiotherapy [51]. The gene GGCT displayed a significant association with embelin sensitivity in this study. It has been implicated in cell proliferation, suggesting its potential as a target for reversing chemotherapy resistance [52,53]. Anti-GGCT siRNA has been demonstrated as a promising strategy for treating resistant MCF-7 breast cancer [54]. Furthermore, we found a strong correlation between EZH2 and imatinib drug sensitivity. Imatinib may have potential applications in prostate cancer treatment [55], as it can induce resistance by recruiting DNMT3A and EZH2 to the promoter region of PTEN in leukemia patients, thereby downregulating the transcription of this gene [56]. These data underscore the significant value of PCDRGs in reversing chemoresistance in tumors.

This study has some limitations. Firstly, the sample size collected from public databases such as TCGA is limited and may not fully represent the heterogeneity of PCa patients. Potential biases in factors such as geographic region, ethnicity, and age may affect the model's generalizability. Secondly, the robustness and reproducibility of the model need further evaluation before clinical application. The clinical significance and benefits of the model warrant further cost-effectiveness analysis. Finally, the potential mechanisms linking PCDRGs to BCR in PCa patients require validation through in vitro and in vivo experiments.

## 5 Conclusion

In summary, this study presents PCDI, a novel 27-gene signature derived from machine learning analysis of multiple PCa cohorts, reflecting programmed cell death pathways. The PCDI-based nomogram robustly predicts 1- to 5-year BCR. Despite limitations, these findings underscore the importance of exploring programmed cell death mechanisms and their interplay with the tumor microenvironment, which may uncover new therapeutic targets and enhance personalized treatment strategies for PCa.

## Ethics declarations

Informed consent was not required because the data used in this study were obtained from public databases.

## Consent for publication

Institutional review board approval and informed consent were not required in the current study because research data are publicly available and all patient data are de-identified.

## Availability of data and materials

All data generated or analyzed during the present study are included in this published article or are available from the corresponding author on reasonable request.

## Funding

This work was supported by the Zhejiang Provincial Science and Technology Projects [Grant number: 2024ZL685, 2024ZR133], Key projects of 10.13039/501100003786Hangzhou Science and Technology Bureau [Grant number: 20231203A12].

## CRediT authorship contribution statement

**Feng Gao:** Writing – original draft, Visualization, Validation, Software, Resources, Methodology, Investigation, Formal analysis, Data curation, Conceptualization. **Yasheng Huang:** Writing – original draft, Visualization, Validation, Software, Resources, Methodology, Investigation, Formal analysis, Data curation, Conceptualization. **Mei Yang:** Writing – original draft, Visualization, Validation, Investigation, Formal analysis, Data curation, Conceptualization. **Liping He:** Visualization, Validation, Formal analysis, Data curation. **Qiqi Yu:** Visualization, Validation, Formal analysis. **Yueshu Cai:** Visualization, Validation, Formal analysis. **Jie Shen:** Writing – review & editing. **Bingjun Lu:** Writing – review & editing, Supervision, Project administration, Funding acquisition.

## Declaration of competing interest

The authors declare that they have no known competing financial interests or personal relationships that could have appeared to influence the work reported in this paper.

## Figures and Tables

**Figure:**
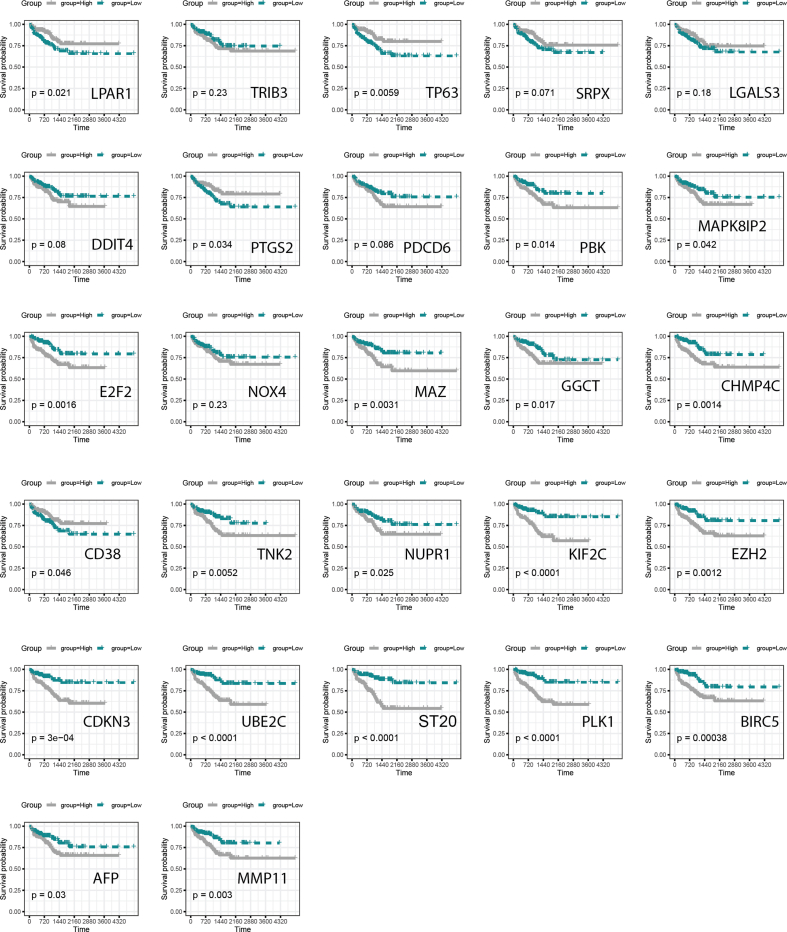
The K-M survival curves for the 27 component genes of the PCDI. Stratification of TCGA-PRAD samples into high and low expression groups was performed according to the median expression level of each gene, followed by survival analysis accordingly.

**Figure:**
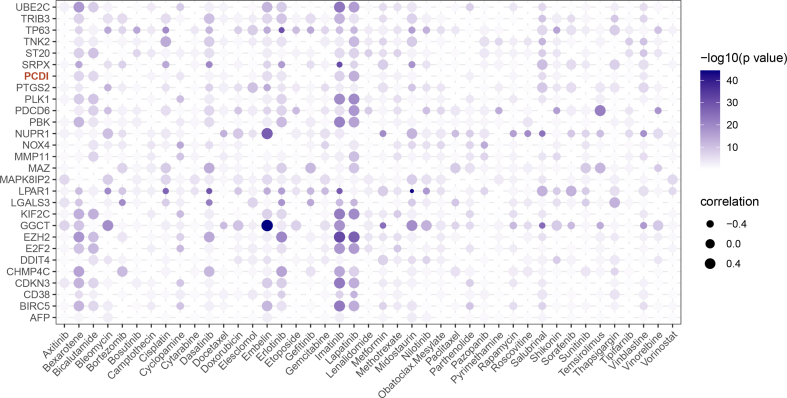
Dot plot illustrating the relationship between the drug sensitivity with PCDI and PCDI-related PCDRGs.

## Abbreviations

PCa: Prostate cancer
PCD: programmed cell death
ML: Machine learning
csPCa: clinically significant PCa
MRI: magnetic resonance imaging
TIME: tumor immune microenvironment
PCDRG: PCD-related genes
BCR: biochemical recurrence
TCGA: The Cancer Genome Atlas
GEO: Gene Expression Omnibus
FPKM: Fragments Per Kilobase of transcript per Million mapped reads
SVM: Support Vector Machine
GBM: Gradient Boosting Machine
LOOCV: leave-one-out cross-validation
ROC: Receiver Operating Characteristic
DEG: differentially expressed genes
GO: gene ontology
KEGG: Kyoto Encyclopedia of Genes and Genomes
GSEA: gene set enrichment analysis
TCIA: The Cancer Immunome Atlas
IPS: immunophenoscores
CCLE: Cancer Cell Line Encyclopedia
SD: standard deviation
CRPC: castration-resistant PCa
TAM: tumor-associated macrophages
CNV: copy number variation
AMP: Amplification
HOMDEL: Homozygous Deletion
RSF: random survival forest
AUC: area under the curve
OS: overall survival
TMB: tumor mutational burden
BP: biological process
CC: cellular component
MF: molecular function
