# Supplementary material for: Machine learning-based cell death marker for predicting prognosis and identifying tumor immune microenvironment in prostate cancer
Source: Heliyon. 2024 Sep 6;10(18):e37554. doi: 10.1016/j.heliyon.2024.e37554 (PMC11414577; doi:10.1016/j.heliyon.2024.e37554)
Supplement: Multimedia component 2 [file mmc2.docx]

**Signature generated from machine learning based integrative approaches**

To develop a PCDRG-related index (PCDI) with high accuracy and stability performance, we integrated 10 machine learning algorithms includingrandom survival forest (RSF), elastic network (Enet), Lasso, Ridge, stepwise Cox, CoxBoost, partial least squares regression for Cox (plsRcox), supervised principal components (SuperPC), generalized boosted regression modeling (GBM), and survival support vector machine (survival-SVM). A few algorithms possessed the ability of feature selection, such as Lasso, stepwise Cox, CoxBoost, and RSF. Thus, we combined these algorithms to generate a consensus model. In total, 101 algorithm combinations were conducted to fit prediction models based on the leave-one-out cross-validation (LOOCV) framework. The initial signature discovery was performed in TCGA-PRAD.The RSF model was implemented via the randomForestSRC package. RSF had two parameters ntree and mtry, where ntree represented the number of trees in the forest and mtry was the number of randomly selected variables for splitting at each node. We used a grid-search on ntree and mtry using LOOCV framework. All the pairs of (ntree, mtry) are formed and the one with the best C-index value is identified as the optimized parameters. The Enet, Lasso, and Ridge were implemented via the glmnet package. The regularization parameter, λ, was determined by LOOCV, whereas the L1-L2 trade-off parameter, α, was set to 0-1 (interval =0.1). The stepwise Cox model was implemented via survival package. A stepwise algorithm using the AIC (Akaike information criterion) was applied, and the direction mode of stepwise search was set to "both", "backward", and "forward", respectively. The CoxBoost model was implemented via CoxBoost package, which is used to fit a Cox proportional hazards model by componentwise likelihood-based boosting. For the CoxBoost model, we used LOOCV routine optimCoxBoostPenalty function to first determine the optimal penalty (amount of shrinkage). Once this parameter was determined, the other tuning parameter of the algorithm, namely, the number of boosting steps to perform, was selected via the function cv.CoxBoost. The dimension of the selected multivariate Cox model was finally set by the principal routine CoxBoost. The plsRcox model was implemented via plsRcox package. The cv.plsRcox function was used to determine the number of components requested, and the plsRcox function was applied to fit a partial least squares regression generalized linear model. The SuperPC model was implemented via superpc package, is a generalization of principal component analysis, which generates a linear combination of the features or variables of interest that capture the directions of largest variation in a dataset. The superpc.cv function used a form of LOOCV to estimate the optimal feature threshold in supervised principal components. To avoid problems with fitting Cox models to small validation datasets, it uses the "pre-validation" approach. The GBM model was implemented via superpc package. Using the LOOCV, the cv.gbm function selected index for number trees with minimum cross-validation error. The gbm function was used to fit the generalized boosted regression model. The survival-SVM model was implemented via survivalsvm package. The regression approach takes censoring into account when formulating the inequality constraints of the support vector problem.
